# Proof of concept of a 45-second cardiorespiratory fitness self-test for coronary artery disease patients based on accelerometry

**DOI:** 10.1371/journal.pone.0183740

**Published:** 2017-09-06

**Authors:** Gabriele Papini, Alberto G. Bonomi, Wim Stut, Jos J. Kraal, Hareld M. C. Kemps, Francesco Sartor

**Affiliations:** 1 Department of Information Engineering, University of Pisa, Pisa, Italy; 2 Department of Electrical Engineering, Eindhoven University of Technology, Eindhoven, The Netherlands; 3 Personal Health Department, Philips Research, Eindhoven, The Netherlands; 4 FLOW Center for Prevention and Rehabilitation of Chronic Diseases, Máxima Medical Center, Eindhoven, The Netherlands; 5 Department of Cardiology, Máxima Medical Center, Eindhoven, The Netherlands; Kurume University School of Medicine, JAPAN

## Abstract

Cardiorespiratory fitness (CRF) provides important diagnostic and prognostic information. It is measured directly via laboratory maximal testing or indirectly via submaximal protocols making use of predictor parameters such as submaximal ${\dot{V}}_{O2}$, heart rate, workload, and perceived exertion. We have established an innovative methodology, which can provide CRF prediction based only on body motion during a periodic movement. Thirty healthy subjects (40% females, 31.3 ± 7.8 yrs, 25.1 ± 3.2 BMI) and eighteen male coronary artery disease (CAD) (56.6 ± 7.4 yrs, 28.7 ± 4.0 BMI) patients performed a ${\dot{V}}_{O2peak}$ test on a cycle ergometer as well as a 45 second squatting protocol at a fixed tempo (80 bpm). A tri-axial accelerometer was used to monitor movements during the squat exercise test. Three regression models were developed to predict CRF based on subject characteristics and a new accelerometer-derived feature describing motion decay. For each model, the Pearson correlation coefficient and the root mean squared error percentage were calculated using the leave-one-subject-out cross-validation method (r_cv_, RMSE_cv_). The model built with all healthy individuals’ data showed an r_cv_ = 0.68 and an RMSE_cv_ = 16.7%. The CRF prediction improved when only healthy individuals with normal to lower fitness (CRF<40 ml/min/kg) were included, showing an r_cv_ = 0.91 and RMSE_cv_ = 8.7%. Finally, our accelerometry-based CRF prediction CAD patients, the majority of whom taking β-blockers, still showed high accuracy (r_cv_ = 0.91; RMSE_cv_ = 9.6%). In conclusion, motion decay and subject characteristics could be used to predict CRF in healthy people as well as in CAD patients taking β-blockers, accurately. This method could represent a valid alternative for patients taking β-blockers, but needs to be further validated in a larger population.

## Introduction

Cardiorespiratory fitness (CRF), or functional capacity, is defined as the ability to perform daily living physical tasks by means of preeminent aerobic metabolic processes [1], and it provides important diagnostic and prognostic information [1]. In sports medicine it is used to predict endurance performance [2], whilst in cardiac rehabilitation it is an important parameter for characterizing the severity of cardiac limitations, prescription of an exercise programs, and evaluating post-event recovery [3, 4]. Moreover, this parameter has been shown to be an excellent independent risk factor for cardiovascular diseases [5, 6].

Cardiorespiratory fitness, also called ${\dot{V}}_{O2peak}$, is traditionally measured directly via laboratory maximal exercise testing, or indirectly via submaximal exercise protocols making use of predictor parameters such as oxygen consumption (${\dot{V}}_{O2}$), heart rate (HR), rating of perceived exertion, and workload [1, 7]. The importance of testing CRF on a large scale was already stressed in a preventive context by the Canadian Physiology Society in the late 70’s [8]. For this purpose a simple submaximal step home test was designed [8]. About the same time, H. J. Montoye deployed another submaximal step home test in his epidemiologic study in order to evaluate exercise capacity of the entire community of Tecumseh, Michigan [9]. Those types of submaximal tests were employed because they were rather inexpensive; they did not need special supervision and they could be performed in a heterogeneous population. However, they still required specific tools such as an exercise step, and around 30 minutes of preparation time [10]; and they are still a tradeoff between increased convenience and reduced accuracy [7].

Nowadays, mobile health has become a growing reality [11, 12]. In cardiac rehabilitation, telehealth interventions have shown to be at least as effective as conventional rehabilitation, with the advantage for the patients to remain in their familiar environment and for the health care system lower costs [13]. In this context it is important to have a safe, reliable, and easy to perform CRF home test, which could be executed by cardiac patients at home using as little extra equipment as possible. We have extensively reviewed submaximal protocols to assess CRF, which could be suitable for the home setting [7]. Most of these submaximal protocols use HR and/or workload to estimate CRF.

However, HR is not a reliable parameter to estimate maximal aerobic capacity in patients on β-blockers [14, 15]. In fact, blockades of β-adrenoceptors slow down HR at rest and attenuate its increase during exercise [16]. The cardiorespiratory and cardiovascular effects of β receptor blockade are more complex than a mere reduction in HR; at pulmonary level, β_2_ receptor inhibition causes bronchoconstriction, while at peripheral vessels level vasoconstriction [17]. Furthermore, myocardial oxygen consumption is reduced by β-blockers [17]. At rest as well as during exercise the effect of chronic β-blockers treatment on ${\dot{V}}_{O2}$ and HR does not seem to be proportional. Gullestad et al. [18] observed no reduction of ${\dot{V}}_{O2}$ at rest, a slight reduction of 2% in ${\dot{V}}_{O2}$ at submaximal level and 7.5% for peak ${\dot{V}}_{O2}$, mainly explained by a lower peak workload, whereas they observed a steady reduction in HR of 28% at rest, 26% at submaximal level, and 27% at peak exercise level when compared with placebo. Furthermore, Wolfel et al. [19] found a striking increase in oxygen pulse (${\dot{V}}_{O2}/\mathrm{HR}$) due to acute and chronic β-adrenergic blockades in both submaximal and maximal exercise conditions. Interestingly, reduction in ${\dot{V}}_{O2max}$ and peak workload were less pronounced in this latter study [19].

It becomes evident that HR-based estimation models would inaccurately estimate CRF on an individual basis, while either workload-based or subjective rating estimation would be more appropriate [14]. However, accurate workload measurements require ad-hoc equipment, such as cycle-ergometers or treadmills, which usually are not available at home. In order to overcome the above mentioned limitations of submaximal testing based on HR and/or workload measurements, we have developed an innovative methodology for predicting CRF from periodic body motion decay. We used a submaximal physical exercise such as repetitive squats executed at a given tempo (i.e. 80 bpm, one beat squat down and one beat stand up) for a time period long enough to increase HR and physical fatigue perception [20]. This protocol was recently validated for the estimation of CRF from HR and subject characteristics in a large population of healthy individuals [20]. Since HR-based estimations would not be suitable for patients on β-blockers, we have hypothesized that CRF could be estimated from the progressive deviation of motion patterns from the ideal motion pattern required. In simple terms: we anticipate that less fit people would deviate from the ideal motion pattern faster and with a greater magnitude, than fitter people. The only device required to carry out this test was an activity monitor (i.e. tri-axial accelerometer), and a metronome.

To the authors knowledge this is the first time that CRF is predicted solely by body motion. The purpose of this study was to prove the idea that body motion information during a periodic movement (e.g. 45 second of squatting) is able to provide CRF prediction in healthy subjects as well as coronary artery disease (CAD) patients.

## Materials and methods

Forty-nine subjects volunteered to take part in our investigation. Thirty were healthy individuals and all of them are included in the data analysis. Nineteen were CAD patients and eighteen of them are included in the data analysis (Table 1, Table 2). The excluded subject was the only woman in the patients group. Her data were not analyzed to avoid misrepresentation of sex in the regression analysis. All patients included in the data analysis, with the exception of three, were on β-blockers. All the subjects recruited were able to perform the physical tasks requested, accordingly to their fitness level. The healthy subjects were recruited in the Eindhoven area via flyers and newspaper advertisements, while the CAD patients were enrolled through the Máxima Medical Center in Veldhoven and Eindhoven. Prior to their participation, all volunteers had time to read the information letter and gave written consent. The protocol of this study was approved by the Internal Committee on Biomedical Experiments of Philips Research as well as by the Medical Ethical Committee of the Máxima Medical Center.

**Table 1:** Subjects’ characteristics.

	n	Weight [kg]	Height [cm]	BMI [kg/m^2^]	Age [years]	R_FSmax_
**Healthy**						
**Female**	12	67.9±8.2***	170.1±4.8***	23.4±2.4*	31.3±8.4	16.2±5.9
**Male**	18	83.7±9.8***^,^^§§^	178.9±5.9***	26.2±3.2*	31.2±7.7^§§§^	20.2±8.5^§§§^
**total**	30	77.4±12^+++^	175.4±7.0^+^	25.1±3.2^++^^,^^§^	31.3±7.8^+++^	18.6±7.7^+++^
**CAD patients**						
**Male**	18	93.7±11.7^+++^^,^^§§^	180.8±6.6^+^	28.7±4^++^^,^^§^	56.6±7.4^+++^^,^^§§§^	4.6±1.8^+++^^,^^§§§^

R_FSmax_ = Maximum cross-correlation between the initial and last parts of the accelerometer signal (explained in detail in the Data Analysis section).

*,*** = significant difference between the two sexes in the healthy group, p<0.05, and p<0.001, respectively.

+,++,+++ = significant difference between the healthy group and the CAD patients group; p<0.05, p<0.01 and p<0.001, respectively.

§,§§,§§§ = significant difference between the male subjects in the healthy group and in the CAD patients group, p<0.05, p<0.01 and p<0.001, respectively.

**Table 2:** Coronary artery disease patients.

Subject	Diagnose	Intervention	β-blocker	dose [mg]	ACE inhibitor	dose [mg]	AR blocker	dose [mg]
1	non STEMI	PCI	Metoprolol	50	Perindopril	4		
2*	suspected AP	Drug treatment	Metoprolol	50	Lisinopril	5		
3	non STEMI	PCI	Metoprolol	50	Perindopril	4		
4	stable AP	CABG	Metoprolol	50				
5	MI	PCI (DES)	Metoprolol	50	Perindopril	2		
6	non STEMI	PCI						
7	non STEMI	Drug treatment	Metoprolol	100	Perindopril	2		
8	AP	PCI	Metoprolol	100				
9	non STEMI	Drug treatment						
10	non STEMI	Drug treatment	Metoprolol	50	Perindopril	4		
11	MI	PCI	Metoprolol	100			Valsartan	160
12	MI	Drug treatment	Metoprolol	50	Lisinopril	5		
13	AP	PCI	Metoprolol	50				
14	non STEMI	CABG	Metoprolol	100				
15	MI	PCI	Metoprolol	50	Enalapril	5		
16	non STEMI	PCI	Metoprolol	100	Perindopril	2		
17*	complains of AP	Drug treatment					Valsartan	320
18	AP	CABG	Metoprolol	100				

STEMI = ST elevated myocardial infarction; AP = angina pectoris, PCI = percutaneous coronary intervention, CABG = coronary artery bypass graft, DES = drug-eluting stent

* Both patients #2 and #17 had documented coronary artery disease. Patient #2 had a PCI and patient #17 had a CABG intervention in their recent history. However, both patients returned to the hospital with suspected AP. Drug treatment was intensified and they were referred to cardiac rehabilitation.

### Cardiorespiratory fitness assessment

Subjects were asked to come to the Máxima Medical Center for a cycle ergometer ${\dot{V}}_{O2peak}$ test. Subjects were instructed to wear comfortable sports clothes and having fasted for the previous two hours from food and caffeine. Upon arrival, they had their weight and height measured. The subjects were then seated on the cycle ergometer and set up for the CRF assessment. The cardiorespiratory fitness was measured using breath by breath metabolic carts (Oxycon Pro Metabolic Cart, Carefusion, California, USA and Masterscreen™ CPX, CareFusion, Hoechberg, Germany). The test was conducted by trained exercise physiologists, who calculated a ramp protocol following ACSM guidelines [21] aiming at a maximum workload being reached after 10 minutes. After a 2 minute warm up at a light intensity, the test began. The load on the cycle ergometer progressively increased every 6 seconds according to the protocol selected by the exercise physiologist. Subjects were given encouragement in order to help them cycle until complete exhaustion. The test ended once the subject could no longer maintain a pedaling cadence above 60 rpm. After completing a 3 minute cool down subject were allowed to stop. ${\dot{V}}_{O2peak}$ was calculated as the final 30 second averaged value of the test.

### Submaximal testing

A few days after the cardiorespiratory fitness assessment, the subjects were requested to do a squat exercise for 45 seconds. Each repetition is composed of two movements, one squatting down and one standing back up, each one executed following the audio feedback of a metronome set at 80 bpm. In literature, this test modality has been found appropriate to assess cardiovascular fitness on healthy subjects using HR and physical characteristics data [20, 22], and therefore it has been considered suitable also for this research. Subjects were instructed to perform a squat as we define it here: bending their knees to create an internal angle between the femur and the tibia of around 90°. During the exercise the tri-axial acceleration was recorded. Patients used a research version of the DirectLife Activity Monitor (DL, range ± 2 g; sampling frequency 20 Hz, Philips Research, Netherlands, Eindhoven) accelerometer placed on the belt. Healthy subjects used a Cardio and Motion Monitoring Module Generation 1 (CM3g1, range ± 8 g; sampling frequency 16 Hz, Philips Research, Netherlands, Eindhoven) accelerometer placed on the wrist.

This type of sensors allow to record the acceleration of body segments where they are placed. In their tri-axial configuration, these sensors can completely capture the movement in the three dimensional space. The tri-axial acceleration signals have been used in literature to describe the motion of the subject in terms of type, quantity and quality [23, 24]. Thus, tri-axial accelerometry is suitable to be used for motion decay quantification. The acceleration signals were uniformed (range ± 2 g; sampling frequency 20 Hz) off-line prior to further analysis.

### Data analysis

**Fig 1:**
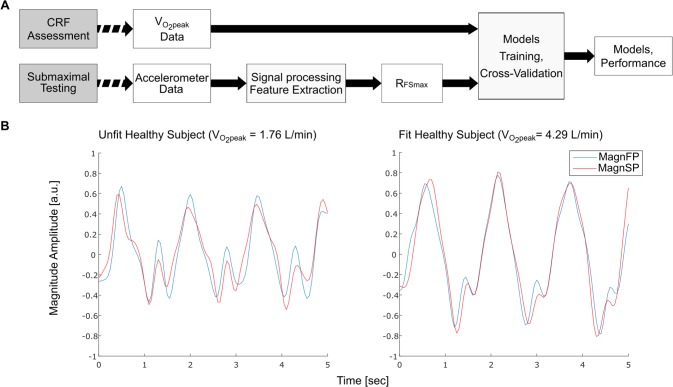
Study workflow and accelerometer output comparison. A) Flowchart describing the main elements of the study B) Examples of 5 seconds segments of the magnitude at the beginning and at the end of the squat exercise in an unfit and a fit representative subject.

The study design and the data analysis flow are diagrammatically represented in Fig 1A. The raw accelerations recorded during the squat test were organized in a database to easily allow feature extraction. Before this operation the acceleration signal from each sensing axis of the sensor (X_acc_, Y_acc,_ Z_acc_) was used to calculate the Euclidean norm, here called magnitude vector of the acceleration signal, as described in this formula:

$$Magnitude=\sqrt{X_{acc}^{2}+Y_{acc}^{2}+Z_{acc}^{2}}$$

The magnitude vector was segmented in two parts of 150 samples each (7.5 seconds), the first from the 200^th^ and the 350^th^ sample (MagnFP, Magnitude First Period) and the second from 650^th^ and the 800^th^ (MagnSP, Magnitude Second Period). In this way the signal irregularities due to the adaptation process of the volunteer to the start of the squat task were removed. The resulting segments of the signals were filtered using a low-pass filter with cut-off frequency of 4 Hz after subtraction of the mean. From the filtered signals in the two segments, the cross-correlation between each couple of MagnFP and MagnSP was calculated (R_FS_). For two discrete time series of data the R_FS_ for the sample n results mathematically:

$$R_{FS}[n]=(MagnFP★MagnSP)[n]≝\sum_{m=-150}^{150}MagnFP[m]\;MagnSP[n+m]$$

The R_FS_ is commonly used in literature to compare signals [25] and also for activity recognition purposes [24]. The maximum of this function (R_FSmax_) is the expression of the maximum similarity between the two segments up to the lag of one respect to the other. Therefore, a higher R_FSmax_ value indicates a lower difference between MagnFP and MagnSP. This feature was selected to represent the ability of the volunteer to maintain a similar motion pattern of moving limbs over time and could reflect the onset of fatigue during squatting. To summarize, when the perceived effort of squatting was high, the similarity between the first and second part of the signal was low. An example of motion decay can be seen in Fig 1B where the healthy subject with normal to low fitness has a higher dissimilarity between MagnFP and MagnSP compared to a healthy fit subject.

The statistical analysis was conducted in Matlab (R2013b, Matworks). The prediction models were built by using stepwise forward multiple linear regressions. Leave one subject out cross-validation was used to evaluate the root mean squared error (RMSE_cv_) of each model, both as absolute value in [L/min] and as percentage respect to the mean ${\dot{V}}_{O2peak}$ of the group. The cross-validation step was employed to evaluate the risk of overfitting and thus to evaluate the overall generalizability of the models. Pearson correlation coefficient (r), adjusted r^2^, bias, limits of agreement were also calculated for each model. Data can be found in the supplementary material S1 Data.

## Results

Using a linear regression technique, three models were created. The first model (Model 1a) was derived and validated on the healthy subjects. The second model (Model 1b) includes a subset of the healthy population, with a fitness level below 40 ml/kg/min. Finally the third model was built on CAD patients ‘data (Model 2). The healthy group and the CAD group were statistically different on most of their anthropometrical parameters (Table 1).

### Motion-based cardiorespiratory fitness models

**Fig 2:**
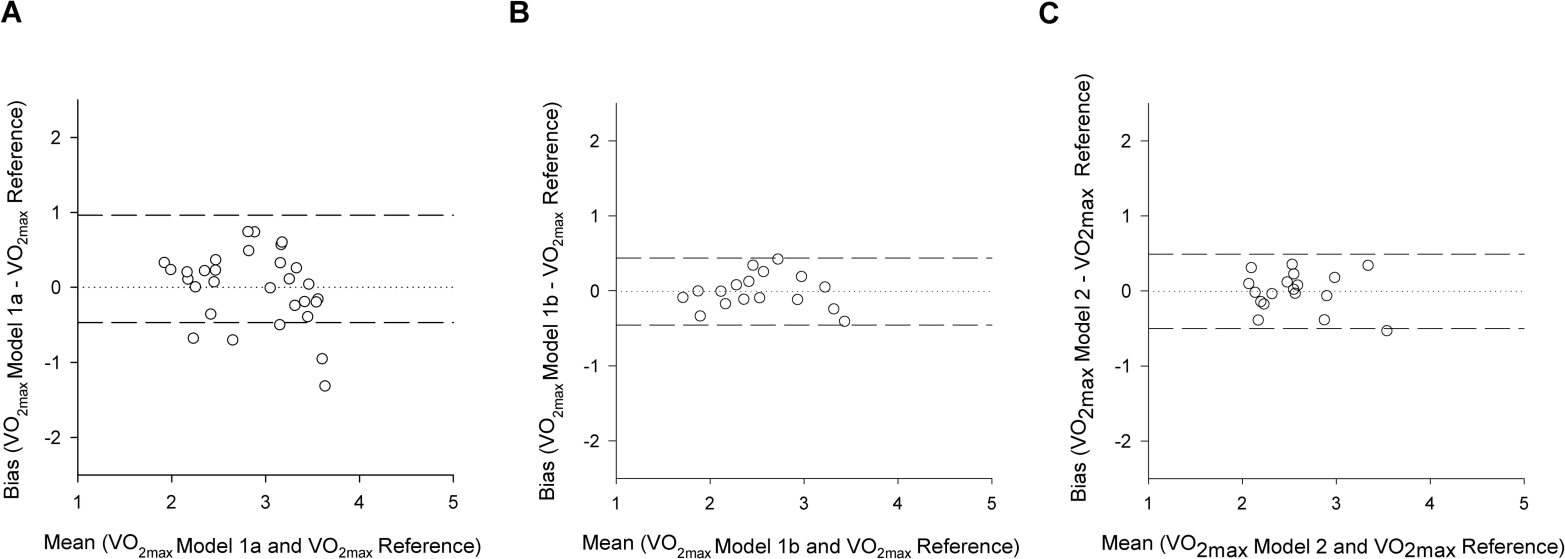
Bland-Altman plots. A) Model 1a, healthy subjects; C) Model 1b, normal to low fitness healthy subjects; D) Model 2, CAD patients. Bias and mean values are expressed in L/min.

All models are described in Table 3. Model 1a included all healthy subjects. The RMSE_cv_ for this model was 0.482 [L/min], equal to 16.7% of the mean ${\dot{V}}_{O2peak}$ measured. The results of its validation process are shown in Fig 2A.

**Table 3:** Multiple linear regression models to predict ${\dot{V}}_{O2peak}$.

	Coef.	SE	t	*p* level	r	Adj.r^2^	RMSE	Bias	LoA	(LOOCV)
							(L·min^-1^)	(L·min^-1^)	(L·min^-1^)	(L·min^-1^)
**Healthy (n = 30)**										
**Model 1a**					0.786	0.556	0.437	0.001	0.962	0.482
									-0.956	
Constant	1.58700	0.725	2.191	0.038						
Body Weight	0.01443	0.009	1.492	0.148						
Age	-0.01759	0.011	-1.616	0.119						
Sex	0.67400	0.238	2.834	0.009						
R_Fsmax_	0.01712	0.012	1.479	0.152						
**Normal to low fitness Healthy (n = 17)**										
**Model 1b**					0.955	0.882	0.183	0.009	0.456	0.221
									-0.437	
Constant	0.14500	0.581	0.249	0.808						
Body Weight	0.02990	0.007	4.352	<0.001						
Age	-0.01820	0.006	-3.157	0.008						
Sex	0.18000	0.169	1.066	0.307						
R_Fsmax_	0.03050	0.008	3.641	0.003						
**CAD (n = 18)**										
**Model 2**					0.914	0.800	0.205	0.005	0.501	0.246
									-0.492	
Constant	4.62400	0.602	7.679	<0.001						
Body Weight	0.00311	0.005	0.672	0.512						
Age	-0.05160	0.007	-7.381	<0.001						
R_Fsmax_	0.12300	0.029	4.166	<0.001						

SE = Standard error, RMSE = root mean square error, LoA = limits of agreement, LOOCV = leave one out cross validation root mean square error

**Fig 3:**
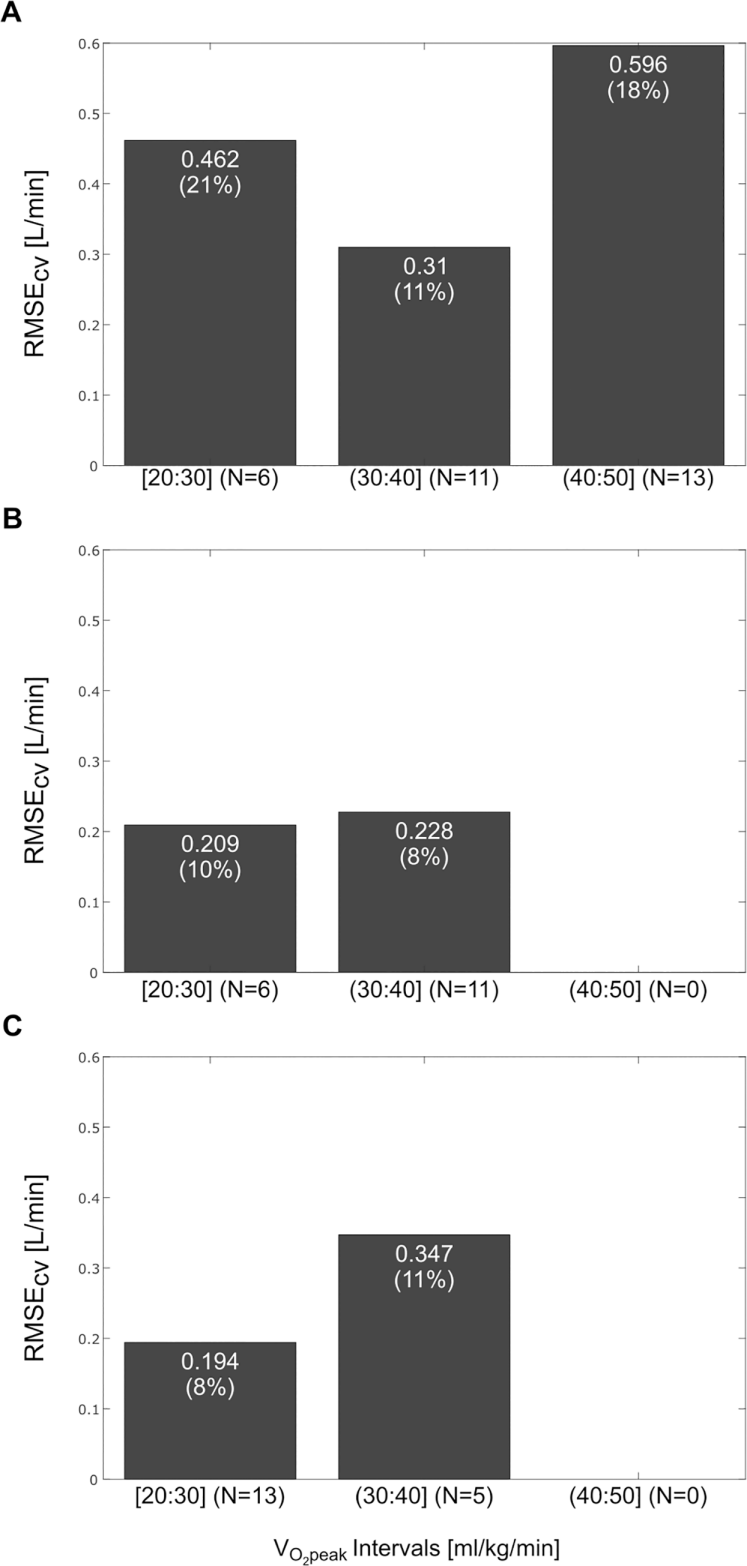
Distribution of the RMSE_cv_ for different fitness categories. A) Model 1a; B) Model 1b; C) Model 2. The values in each bar represent the RMSE_cv_ in [L/min] and, in parenthesis, the RMSE_cv_ in percentage respect to the average ${\dot{V}}_{O2peak}$ of the fitness category.

The healthy subjects were classified in three fitness groups, ranging from 20 to 50 [ml/kg/min] with an increase of 10 [ml/kg/min], to reveal any dependency between the error and the fitness level. The RMSE_cv_s per category are reported in Fig 3A. The group with fitness above 40 [ml/kg/min] showed an overall higher error than the other groups, almost a twofold RMSE_cv_ compared to the central fitness category, probably because the fitness level influenced the results of the model proposed.

Starting from the assumption that high cardiovascular fitness could influence the predictive power of the predictors, especially the R_FSmax_, a second model (Model 1b) was created based on normal to low fitness subjects with a ${\dot{V}}_{O2peak}$ normalized by weight lower than 40 [ml/min/kg] (The RMSE_cv_ for Model1b was 0.221 [L/min], equal to 8.7% of the average ${\dot{V}}_{O2peak}$ measured (Fig 2B). For Model 1b the RMSE_cv_ analyzed for fitness categories did not show any relation between fitness level and error (Fig 3B). Finally, Model 2 was obtained considering all male CAD patients. The term related to the sex is not present because of the uniformity of the considered subjects (all male). The RMSE_cv_ for this model was 0.246 [L/min], equal to 9.6% of the mean measured ${\dot{V}}_{O2peak}$ (Fig 2C). For Model 2, the RMSE_cv_ difference between the highest and the lowest fitness categories was clear, but the two categories presented a similar RMSE_cv_ in percentage (11% versus 8%), therefore it was safe to assume that there is no influence of the fitness level on the error (Fig 3C).

## Discussion

This study presents the first evidence that CRF can be predicted by models based on accelerometry data only, gathered during a submaximal exercise test with some additional subject characteristics (i.e. weight, age, sex), in healthy individuals as well as in CAD patients on β-blockers. This innovative methodology makes use of a motion sensor only, a three axial accelerometer, and no additional equipment, rather than a metronome and a stopwatch, basic features of any smartphone.

Most of the existing submaximal models require HR information often accompanied by body movement information, such as workload, speed, and distance covered in a given time [26–28]. Recently also the use of accelerometers has been exploited for cardio-fitness estimation, however, still in combination with HR information [29, 30]. Despite the fact that there are several maximal as well as submaximal tests that estimate ${\dot{V}}_{O2max}$ not taking into account HR, all these models did not make use of accelerometer information, but rather parameters such as speed and subject’s characteristics, [31–33].

In the current study we have observed that sustained physical aerobic activity administered as repetitive squat exercise [20], has determined a fatigue-induced deterioration in the motion patterns. Motion patterns contain information related to the range of motion as well as the movement economy (i.e. ${\dot{V}}_{O2}/\mathrm{displacement}$). We used R_FSmax_, an index of signal similarity, to describe variations in those motion patterns over time. The degree of failure on an optimal physical exercise task execution, expressed as R_Fsmax_, was related to aerobic capacity. This means that a person with high CRF has a minimal failure on this aerobic task, thus a small R_Fsmax_, maintaining a similar motion pattern throughout the exercise test. Conversely, an unfit person shows a greater change in motion pattern between start and end of the exercise test.

We selected an aerobic submaximal exercise test in order to validate our hypothesis that motion pattern deterioration could reflect aerobic capacity [20]. Although this was not tested in the present study, we suggest that this approach could also work for other aerobic protocols, as long as a repetitive exercise pattern is employed, such as stepping at a given pace (e.g. Queens college step test 24 steps/min [34]). This approach could also be applied to repetitive anaerobic tests, such as the repeat jump test [35], as long as the movement frequency is fixed, so that R_Fsmax_ can express task failure.

The model built on all healthy subjects (Model 1a), using R_Fsmax_ as accelerometry feature, and weight, age and sex as subject characteristics, had a comparable RMSE_cv_ to what we observed for the same squat test when HR features and subject characteristics were used (16.7% versus 16.8% respectively) [20]. As shown in the Bland-Altman plot in Fig 1A, there is no significant bias between the measured and the predicted ${\dot{V}}_{O2peak}$. The accuracy, in terms of RMSE_cv_, of Modal 1a is on par with other well established submaximal protocols developed in healthy people, using HR, such as the Rockport walk test proposed by Kline (12.6%), and Rockport walk test modified for treadmill use (15%), the ACSM cycling test (15.5%) [28, 36, 37]. Our accuracy results are comparable also to more recent prediction models based on activity monitoring, such as the Activity Counts over HR in free living proposed by Plasqui & Westerterp (14.1%) [38]. However, Model 1a suffers from the fact that people with a measured CRF above 40 [ml/kg/min] may be aerobically less challenged by a 45 s squat test than people with a lower fitness (Fig 3A). Thus, when we created Model 1b for normal to low fitness people only (${\dot{V}}_{O2peak}$ < 40 [ml/kg/min]), the RMSE_cv_ was 8% lower than in Model 1a. The accuracy of Model 1b, even though only in normal to low fitness subjects, is comparable also to more elaborated fitness estimation algorithms, such as the one proposed from Altini et al. (11.3%) based on activity classification and HR monitoring [30]. Also in this case the model did not show a significant bias (Bland-Altman plot in Fig 2B). Interestingly, the partial correlation coefficient between R_Fsmax_ and ${\dot{V}}_{O2peak}$ increases by circa 120% when excluding fit subjects. Moreover, by removing the fit subjects, the RMSE_cv_ became comparable between fitness categories (Fig 3B). Therefore, we suggest that the squatting protocol should be prolonged for fitter people until a significant motion pattern alteration would be observed (Table 4).

**Table 4:** Partial correlation between ${\dot{V}}_{O2peak}$ and the different predictors (x = predictor not used).

Model	N	Weight	Age	Sex	R_FSmax_
**Model 1a**	30	0.29	-0.31	0.49	0.28
**Model 1b**	17	0.78	-0.67	0.29	0.72
**Model 2**	18	0.18	-0.89	x	0.74

Partial Correlation expresses the correlation between the dependent variable (${\dot{V}}_{O2peak}$) and one of the independent variables (Weight, Age, Sex, R_FSmax_) upon removing the linear effects of the remaining independent variables.

We have applied the same approach to estimate CRF (i.e. ${\dot{V}}_{O2peak}$) in CAD patients on β-blockers using acceleration data only (Model 2). The accuracy of Model 2 was of comparable magnitude (RMSE% = 9.6) to Model 1b, namely normal to low fitness healthy people. No systematic over- or under-estimation was observed. The consistent low error seen in CAD patients as well as normal to low fitness healthy people obtained with our motion-based approach could be explained by the appropriateness of the protocol selected. In fact, CRF levels of these two populations in our study were similar (normal to low fitness = 31.8 ml/kg/min; CAD patients = 27.6 ml/kg/min), although patients had a significantly lower fitness than normal to low fitness healthy individuals (p = 0.006). We hypothesize that the protocol length may need to be adjusted according to the expected fitness level of the users. In our study accelerometry-based CRF estimation was working better in unfit than fit people; this is probably due to the fact that a greater deviation from the optimal physical task execution was measurable in unfit people. Possibly, R_FSmax_ could be used to decide when to stop the exercise because of a significant alteration in movement patterns is achieved.

This was the first attempt to estimate CRF by accelerometry information only; and the authors are aware of some limitations of this study. Although in total we have a sample size of 48 subjects, only 18 were patients. Yet this is a proof of concept study, which aimed to show the potentials of our new methodology. Larger studies should be performed in the future in order to strengthen our results. Confounding factors such as the level of musculoskeletal impediments, the level of execution experience, and the motoric skills were not controlled in this study. Yet, the higher variability expected in our heterogeneous sample did not hamper the statistically significant relation between the movements pattern deterioration and the CRF levels. Another, limitation is that, by chance, the patients who volunteered to participate in this study were all males. Thus, it is yet to be determined how our approach would perform in female patients, considering that a new model, including sex as predictor, should be calculated for the patient group. We do not expect that the performance in female patients would differ much from the performance observed in this study in female healthy individuals.

In conclusion, this research showed that CRF can be predicted with a simple squatting exercise in healthy people as well as CAD patients taking β-blockers solely by using accelerometry and individual’s characteristics (e.g. body weight, sex and age). Further research is needed to optimize test duration according to fitness level, create models for different wearing positions of the accelerometer, and to validate this approach for anaerobic power estimation.

## Supporting information

S1 Data Supporting infomration data_squat.xlsx. (XLSX)
